# Routine ^18^F-FDG PET/CT does not detect inflammation in the left atrium in patients with atrial fibrillation

**DOI:** 10.1007/s10554-017-1094-2

**Published:** 2017-02-22

**Authors:** Philipp S. Lange, Nemanja Avramovic, Gerrit Frommeyer, Kristina Wasmer, Christian Pott, Lars Eckardt, Christian Wenning

**Affiliations:** 1 Division of Clinical and Experimental Rhythmology, Department of Cardiology and Angiology, University Hospital Münster, Albert-Schweitzer-Campus 1, Building A1, 48149 Münster, Germany; 2 Department of Nuclear Medicine, University Hospital Münster, Münster, Germany; 3 Sonderforschungsbereich 656, Münster, Germany

**Keywords:** Atrial fibrillation, Inflammation, Positron emission tomography, Cardiac imaging

## Abstract

Increasing evidence supports a role of inflammation in the development of atrial fibrillation (AF). However, direct evidence of persistent inflammatory activity in the atria of AF patients is scarce. In this study, we used 18-Fluor-Deoxyglucose positron emission tomography computed tomography (^18^F-FDG PET/CT) to determine atrial inflammation in patients with and without AF. Retrospectively, ^18^F-FDG PET/CT scans were analyzed. 37 patients with a history of AF were compared to an age and sex matched control group with no history of AF. Standardized uptake values were obtained in the atrial walls, in the left ventricular wall, and in the right ventricular blood pool, respectively. Target to background ratios (TBR) were determined in the atrial and left ventricular walls and compared between the two groups. TBR values of the left atrial wall were slightly but not significantly higher in patients with AF (1.21 ± 0.27) compared to those without AF (1.14 ± 0.29; p = 0.85). Likewise, a weak but not significant difference was observed in signal intensities in the right atrial wall between patients in the AF (1.14 ± 0.45) and the control group (0.96 ± 0.2; p = 0.41). TBR values of the left ventricular myocardium did not differ between the groups; no significant correlation was found between the TBR in the left and right atrial wall and blood glucose levels. ^18^F-FDG PET/CT performed under routine conditions did not detect a significant difference in inflammatory activity in the left or right atrium between patients with and without AF. Contrary to previous reports, these results therefore do not clearly support a role for ongoing atrial inflammation in patients with AF. Prospective clinical studies using myocardial glucose uptake suppression strategies may be helpful to clarify these issues.

## Introduction

Atrial fibrillation (AF) is the most common arrhythmia in industrialized countries with an increasing burden of morbidity [1–3]. Electrical and structural remodelling of the atria promotes the development, maintenance and progression of AF [4–6]. Remodelling in AF is accompanied by atrial fibrosis, changes in cardiac autonomous innervation and inflammation. Different imaging strategies have been employed to visualize atrial fibrosis and cardiac autonomous innervation. These imaging approaches have shown to provide additional information on the individual risk of AF recurrence and therapy outcome. Imaging of atrial fibrosis [7] and molecular imaging of autonomous innervation [8] might in fact be helpful to better select patients for antiarrhythmic therapies. Inflammation is less well characterized in AF. However, clinical evidence suggests a causative link between inflammation and the occurrence of AF. A number of inflammatory markers including C reactive protein (CRP) and Interleukin-6 have been shown to correlate with the incidence and recurrence rates of AF [9–11]. For instance, C reactive protein (CRP) has been demonstrated to correlate with the incidence of AF and the recurrence after cardioversion [12, 13].

Based on these observations, it has been speculated that inflammation based therapies could even offer novel therapeutic options to patients suffering from AF [14, 15]. However, direct evidence of persistent inflammatory activity in the atria of AF patients is scarce. 18-Fluor-Deoxyglucose positron emission tomography (^18^F-FDG PET) has been successfully employed to visualize inflammatory processes. Data from a small clinical study showed a higher inflammatory activity in adipose tissue determined by PET/CT imaging in patients with paroxysmal AF compared to patients without atrial fibrillation [16]. Mazurek et al. [16] demonstrated higher standardized uptake values (SUV) of the epicardial adipose tissue in the roof of the left atrium, in the atrioventricular groove and in the left main artery of patients with AF compared to the control group. Moreover, Kusayama et al. [17] claimed a higher inflammation activity of the epicardial adipose tissue around the left atrium in patients with atrial fibrillation compared to a control group without AF based on density measurements using cardiac computed tomography. Accordingly, we hypothesized that patients with AF have a higher inflammation activity in the atrial walls that could be detected and quantified by PET/CT imaging. Here, we describe a series of 37 patients with AF and 37 control patients without AF who underwent PET/CT imaging for other clinical indications.

## Patients and methods

We conducted a retrospective single-center study including ^18^F-FDG PET/CT examinations performed between April 2009 and September 2013 with a total of 74 patients. PET/CT scans of patients referred to the Department of Nuclear Medicine for various clinical indications were analyzed. Based on ICD codes, all consecutive patients with a history of AF (n = 37 patients, 25 men; mean age 70 ± 7 years; 21 patients with malignancies, 16 patients with systemic inflammation) were selected and compared to an age and sex matched control group with no history of AF (n = 37 patients, 24 men; mean age 71 ± 7 years; 34 patients with malignancies, three patients with systemic inflammation). Among the patients in the AF group, 24 individuals suffered from paroxysmal forms of AF, 12 patients had chronic forms of AF (persistent or permanent AF). Three patients in the AF group and three patients in the control group suffered from Diabetes, four patients in the control group and eight patients in the AF group received an anti-inflammatory medication at the time of the PET/CT scan. Dyslipidemia was present in 12 patients of the AF group and in two patients of the control group. A diagnosis of arterial hypertension was documented in 13 patients in the control group, while 20 patients in the AF group had a diagnosis of arterial hypertension.

The study was approved by the University of Münster Ethical Committee and Institutional Review Board. Therefore, it has been performed in accordance with the ethical standards laid down in the 1964 Declaration of Helsinki and its later amendments. All patients gave their written informed consent for the PET examination.

## Patient preparation and PET/CT examination

Details concerning the PET/CT procedures have been published elsewhere [18, 19]. To suppress physiological myocardial glucose uptake, all patients were studied after fasting for at least 6 h. Blood glucose levels at the time of ^18^F-FDG application ranged from 76 to 172 mg/dL (mean 110 ± 21 mg/dl). A body-weight—adapted activity of ^18^F-FDG (5 MBq/kg of body weight) was injected intravenously approximately 60 min before PET data acquisition (mean activity: 339 MBq ± 61 MBq, range 205–491 MBq). The scans were obtained using a hybrid PET/CT system (Biograph Sensation 16; Siemens Medical Solutions). Low-dose CT of the entire area covered by PET (from skull base to the mid thigh level) was performed for attenuation correction in all patients. CT scans were obtained either on a 16-slice integrated in the PET/CT system, with a slice thickness of 1.0 or 0.75 mm. After completion of the CT scan, PET data were acquired for 3 min per bed position. PET images were reconstructed with attenuation correction using the standard manufacturer-supplied software.

All PET/CT scans were reviewed and analyzed by two nuclear medicine physicians, experienced in PET analysis, who were not aware of the diagnosis of atrial fibrillation. Image analysis was performed with the Siemens syngo.via software, version 2. ^18^F-FDG uptake was measured using the function Region-Of-Interest (ROI). ^18^F-FDG uptake was evaluated quantitatively by the calculation of standardized uptake values (SUVmax and SUVmean) in the attenuation-corrected images.

**Fig. 1:**
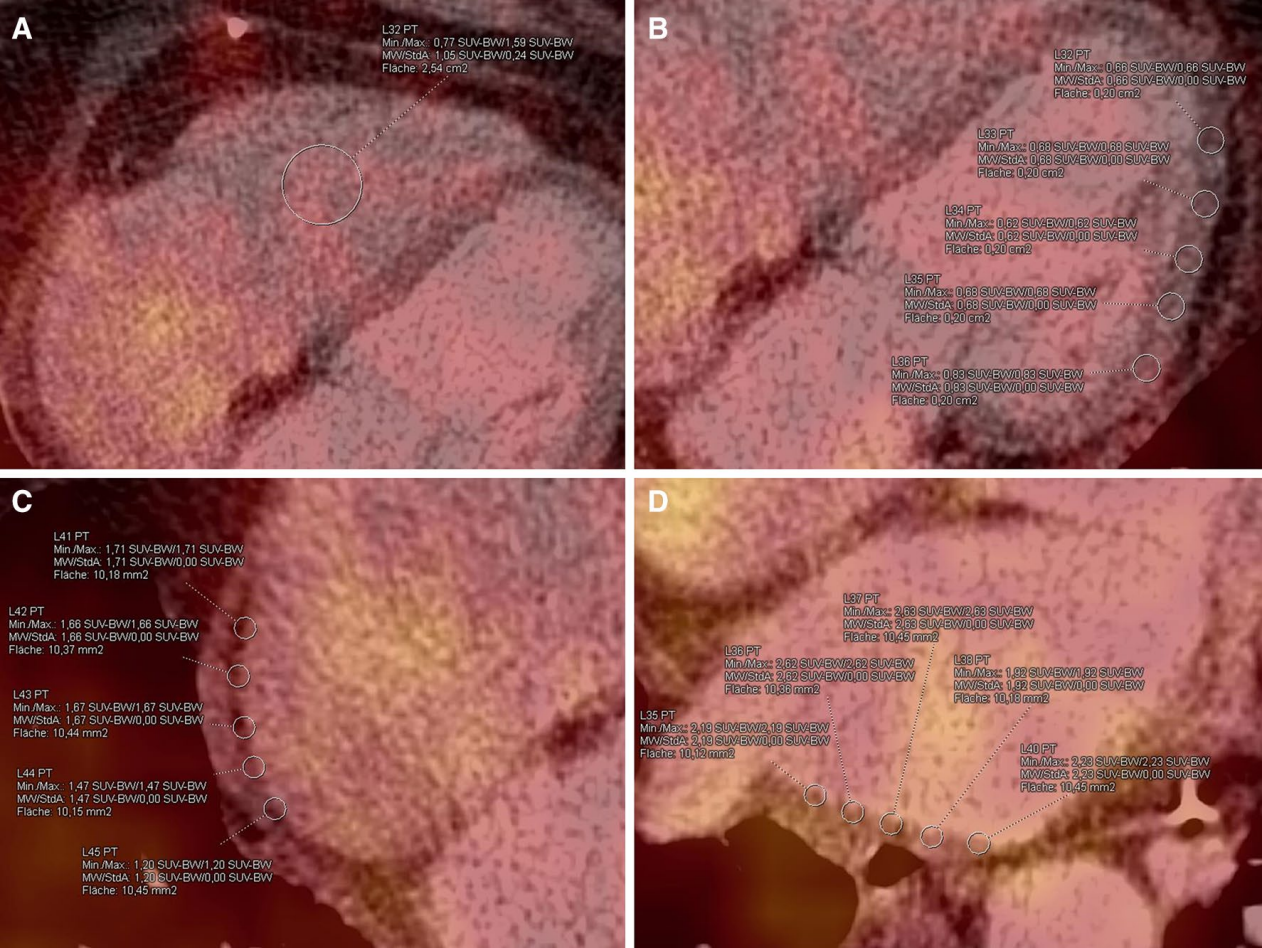
Representative images with selection of ROIs: **a** blood pool within the right ventricle; **b** in the lateral left ventricular wall; **c, d** in the atrial walls

SUVs were obtained in the left and right atrial wall by placing 5 regions of interest (ROI) of equal size (10 mm^2^ each) within the atrial walls, 5 ROIs (20 mm^2^ each) in the left ventricular wall and one ROI (250 mm^2^) in the right ventricular blood pool (Fig. 1). Special care was taken in excluding ^18^F-FDG uptake of neighboring organs. CT images were used for attenuation correction of the PET data and for anatomical correlation.

In order to correct for blood pool activity, target to background ratios (TBR) were determined in each patient by calculating the ratio of the mean of the 5 SUVmax in the atrial wall or the mean of the 5 SUVmax in the left ventricular wall, respectively, and the SUVmean of the right ventricular blood pool.

Continuous variables are presented as mean ± standard deviation, and categorical variables are expressed as frequencies. Pearsons correlation coefficient was calculated for the evaluation of significant association between two parameters. A Kolmogorov–Smirnov test was performed for continous variables to test for normality. A Mann-Whitney-U-test was conducted to compare two means, whereby statistical significance was assumed at p value < 0.05.

Data analysis was performed using SPSS 15.0 software (IBM Corp, Armonk, NY) and Excel 2010 software (Microsoft Corp, Redmond, WA).

## Results

**Fig. 2:**
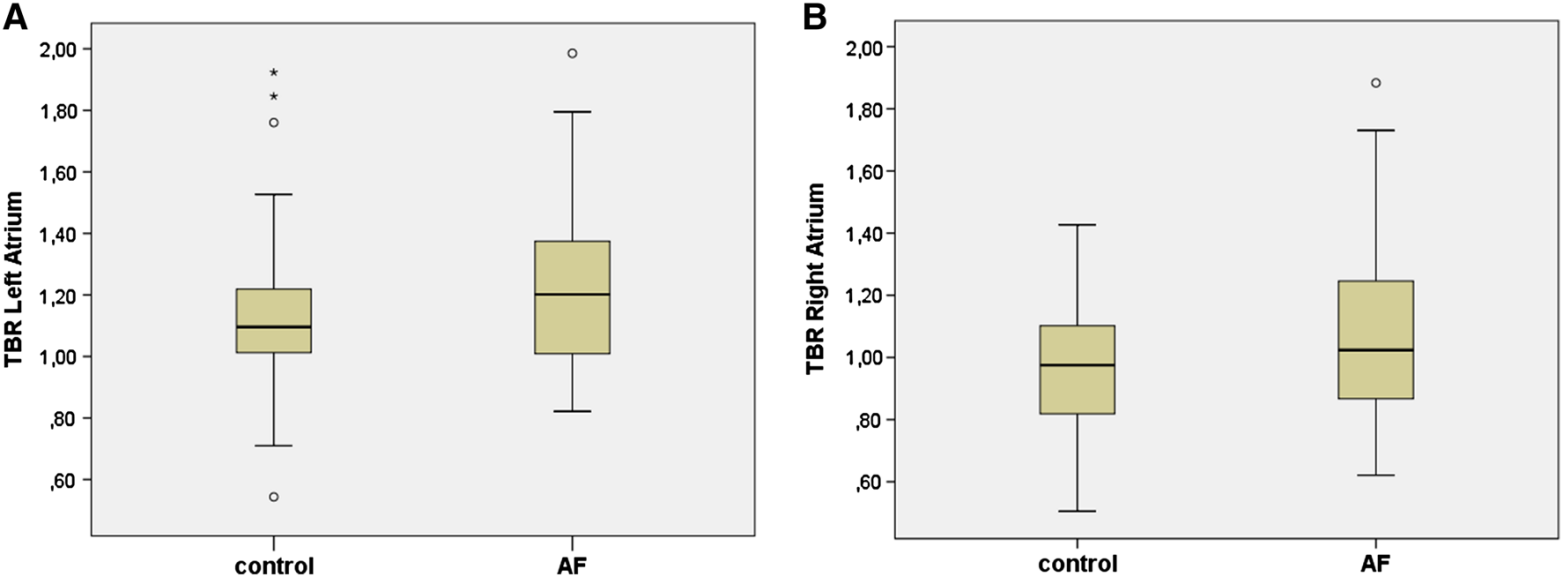
Target to background ratios of patients with and without atrial fibrillation of the *left* (**a**) and *right* (**b**) atrial wall shown as *box plots*

TBR of the *left* atrial wall were slightly but not significantly higher in patients with AF compared to those without AF TBR left atrium 1.14 ± 0.29 (control group) and 1.21 ± 0.27 (AF group, respectively; p = 0.85; Fig. 2a). A weak, albeit not significant difference was observed in signal intensities in the *right* atrial wall between patients in the AF and control group (TBR right atrium 0.96 ± 0.2 (control group) and 1.14 ± 0.45 (AF group, respectively; p = 0.41; Fig. 2b)). TBR values of the left ventricular myocardium did not differ between the groups (p = 0.51).

**Fig. 3:**
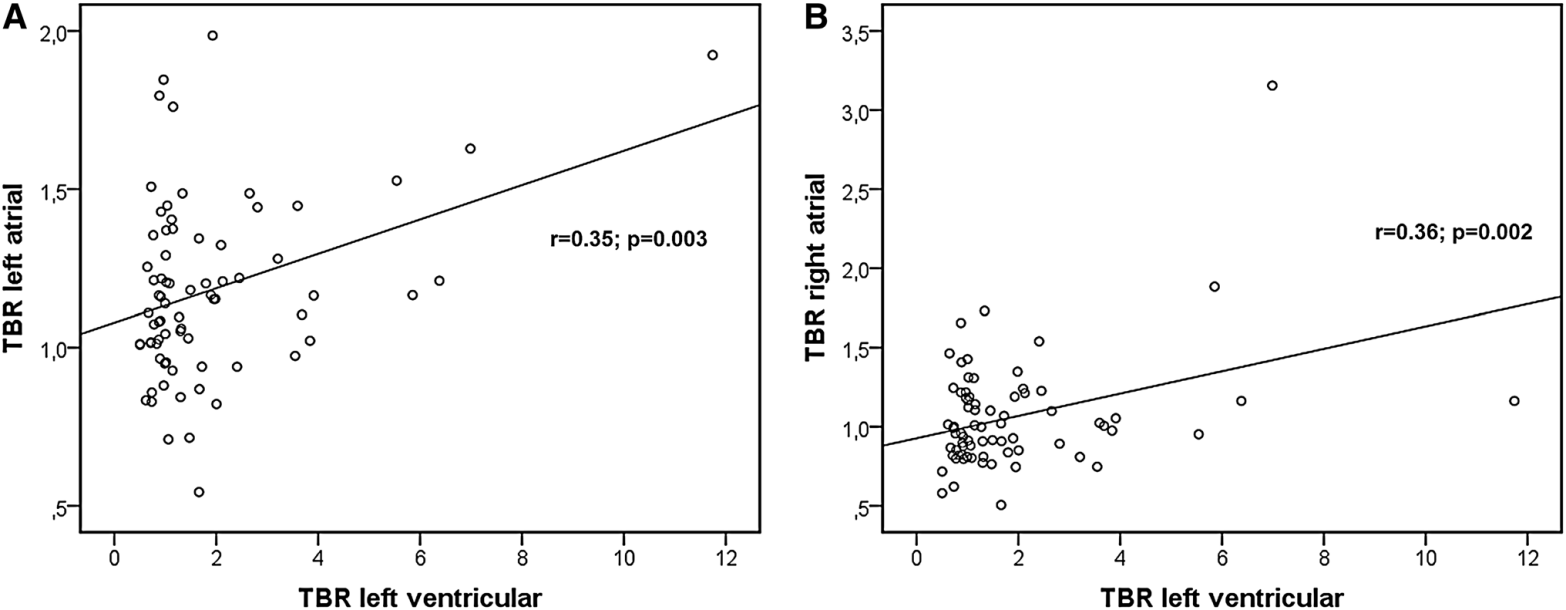
Regression plots of the TBR of the atrial walls vs. TBR of the left ventricular myocardium: **a** left atrial wall; **b** right atrial wall

In both groups, TBR of the left and right atrial walls showed a low to moderate correlation with TBR of the left ventricular myocardium (r = 0.35 and 0.36; p = 0.003 and p = 0.002, respectively; Fig. 3).

In order to investigate metabolic effects that could conceal differences in signal intensities between the control and the AF group, we further analyzed serum blood glucose values. Serum glucose levels were comparable among both groups (113 ± 23 mg/dl (control) vs. 106 ± 16 mg/dl (AF)). No significant correlation was found between the TBR in the left (r=−0.19; p = 0.12) and right (r=−0.12; p = 0.34) atrial wall and the blood glucose levels.

To rule out systemic inflammation as a potential confounder, patients with systemic inflammation were excluded in both groups and tumor patients (n = 21 (AF group) and n = 34 (control group)) were analyzed separately. Again, TBR values of the *left* atrial myocardium did not differ significantly between the groups (TBR left atrium 1.14 ± 0.30 (control group) and 1.25 ± 0.30 (AF group, respectively), p = 0.22). Likewise, the TBR values of the *right* atrial myocardium did not display a significant difference (TBR right atrium 0.96 ± 0.19 (control group) and 1.21 ± 0.53 (AF group, respectively), p = 0.12; not shown). Moreover, in order to decipher a possible association between the duration of atrial fibrillation and the inflammatory activity measured in the atria, patients with paroxysmal and chronic forms of AF were compared. However, a significant difference could not be observed (TBR left atrium 1.22 ± 0.27 (paroxysmal AF) vs. 1.18 ± 0.29 (chronic forms of AF), p > 0.05).

## Discussion

In this retrospective study, we observed a slightly higher ^18^F-FDG uptake in the atrial walls of patients with AF. The direct comparison of atrial TBR values between the AF and control group did not reach the level of statistical significance. It cannot be ruled out that the study of a larger cohort of AF and control patients would reveal statistically significant differences. However, the small absolute differences in ^18^F-FDG uptake argue against the presence of relevant atrial inflammation in the patients of the AF group. Therefore, the PET/CT imaging data presented here do not clearly support an association between a higher inflammatory activity and AF. However, the patients analyzed in this study were referred to the clinic for various indications, mostly malignancies and infections of unknown origin. For this reason, the patient collective in this retrospective study does not fully represent the population of AF patients. However, after exclusion of patients with systemic inflammation, signal intensities in the atrial walls still did not display a significant difference.

Another limitation of the study is the lack of ECG data at the time of the scan. In fact, a time dependent association between inflammation and the onset of atrial fibrillation cannot be ruled out. However, there was no significant difference in the inflammatory activity measured by PET/CT when comparing patients with paroxysmal and chronic forms of atrial fibrillation.

Several converging lines of evidence suggest an important role of inflammation in the complex chain of events eventually leading to the development of AF [20]. It is assumed that local inflammatory processes as well as systemic inflammation can foster the development of AF. Arterial hypertension, obesity, and coronary artery disease have been discussed as sources of inflammation in patients with AF. In animals, arterial hypertension has been shown to promote atrial leucocyte infiltration, inflammation, atrial fibrosis and increased vulnerability to AF suggesting a relationship between these factors [21]. Patients with obesity seem to have a higher risk for progression of AF [20]. Consistently, the pericardial adipose tissue has been shown to promote inflammation and atrial fibrillation [16, 22]. Moreover, AF itself seems to promote inflammation [20, 21] and immune cells have been detected in the atria of patients with AF [20]. In fact, detecting inflammation in AF patients would help to develop tailored anti-inflammatory strategies for the treatment and prevention of AF. Specifically, it could be determined whether specific AF patient subgroups could benefit from anti-inflammatory therapies.

PET(/CT) is an established imaging modality to detect local inflammation. However, the local detection of inflammation by PET critically depends on the concentration and metabolic activity of immune cells within a given tissue volume. Moreover, PET imaging has a limit of spatial resolution [23]. In addition, both cardiac and respiratory motions further impair the spatial resolution, increase image noise and reduce PET signal. Therefore, low level inflammation in the atria and the surrounding tissues might not be detected adequately by PET/CT, and inflammatory “hot spots” might not be evenly distributed around the left or right atrium. Here, in order to record the complete inflammatory activity around the atria, numerous ROIs of equal size were used to determine myocardial tracer uptake. However, very small areas with higher inflammatory activity might still be missed by this image analysis approach. In addition, partial volume effects can influence quantitative PET-analysis of small or thin structures. However, modern PET scanners as used in our study have a spatial resolution of up to 2 mm. Spill-over from the adjacent blood pool could be virtually excluded by CT-guided ROI placement. In fact, PET/CT has already been employed to detect inflammatory activity in AF patients. Mazurek et al. [16] claimed an association of pro-inflammatory activity of epicardial adipose tissue to the occurrence of AF. Of note, in contrast to our investigation, SUV measurements were not corrected for blood pool activity and small single ROIs were used in that study. Moreover, the selection of ROI placement did not follow clear criteria. In our study, the activity in the atrial walls moderately correlated with the activity in the left ventricular myocardium. This observation suggests a generally increased myocardial glucose metabolism rather than a genuinely higher inflammatory activity in patients with AF.

Taken together, contrary to previous results, the present data do not indicate a diagnostic value of ^18^F-FDG PET for the detection of inflammatory activity in the left and right atria of patients with a history of AF when performed under routine conditions. Generally, the detection of inflammatory activity in the atria may be hampered by the physiological myocardial glucose metabolism. Thus, prospective clinical imaging studies of a larger homogeneous cohort of AF patients in the absence of systemic disease and relevant comorbidities using dedicated techniques for the suppression of physiological myocardial glucose uptake such as preparation with a “high fat low carb” diet and/or the administration of heparin prior to PET scanning should be considered.
